# Constructs, Predictive Factors and Measures: Insights From the Mental Health in the Moment (MHIM) Co‐Production Panel

**DOI:** 10.1111/hex.70686

**Published:** 2026-05-16

**Authors:** Luke Power, Dejla Hoxha, Lorna Caddick, Tong Xie, Hannah Coulstock, Alex Crocker, Katie Dryburgh, Daria Melashenko, Dacian Astilean‐Style, Heather Bryson, Eva Drummond, Sharon Ehimen, Poppy Fairbairn, Rowan Kelly, Paige Sinclair, Yu‐Wen Tan, Aja Murray

**Affiliations:** ^1^ Psychology Department, School of Philosophy, Psychology and Language Sciences University of Edinburgh Edinburgh UK; ^2^ Division of Psychiatry, Centre for Clinical Brain Sciences University of Edinburgh Edinburgh UK; ^3^ Faculty of Psychology Beijing Normal University Beijing People's Republic of China; ^4^ Expert by Experience, Member of the ‘Mental Health in the Moment Young Person Advisory’ Group (MHIM‐YPAG)

## Abstract

**Introduction:**

There is growing recognition of the methodological benefits of co‐producing adolescent mental health research. Co‐production supports the development of study methodologies that are more relevant and appropriate for the examination of adolescents' mental health.

Within this article, insights from the Mental Health in the Moment Young Person Advisory Group (MHIM‐YPAG) are presented in relation to which mental health constructs, predictive factors and measures were endorsed by young people for inclusion in a mental health study. The aim of this paper is to provide guidance to researchers, highlight the benefits of co‐production and provide a template for co‐production in mental health study measures.

**Methods:**

Members (*n* = 18) took part in online ‘focus group style’ sessions covering constructs, predictive factors and measures related to adolescent mental health. They were asked to discuss and then individually rank a set of constructs, predictive factors and measures from most to least relevant. Following this, a semantic thematic analysis of session materials was performed by researchers and findings were verified by MHIM‐YPAG members.

**Results:**

Members emphasised the importance of general and specific constructs that reflected both positive and negative traits. In addition, they emphasised the importance of examining suicidality/suicide‐ideation. Social media, academic stress and relationships were the most relevant predictive factors for this group. Language use and response scale length were important when deciding measures appropriateness.

**Conclusion:**

This paper highlights the importance of involving young people as co‐producers within adolescent mental health research. Benefits include improving the appropriateness and relevance of the study design. Insights may act as a guide for researchers involved in adolescent mental health research.

**Patient and Public Contribution:**

The ‘MHIM‐YPAG’ was established to enable the involvement of young people to co‐produce the MHIM project. Thus, this paper details the insights made by the MHIM‐YPAG regarding the inclusion of specific mental health constructs, predictive factors and measures. As detailed in the MHIM‐YPAG protocol paper, young people will be involved through the 5‐year project in a co‐production capacity.

## 1 Background

### 1.1 Adolescents' Mental Health

There has been an observed increase in mental ill‐health within the global north [1], with the majority of mental health issues manifesting before age 24 [2]. Adolescence is a significant period for mental health, with the onset risk of internalising conditions being significant [3, 4, 5, 6]. According to the 2022 Health Behaviours in School‐Aged Children Report, within Scotland, 32% of young people experienced anxiety, and 14% were at risk of depression [7]. Thus, there remains an urgent need for research into mental health during adolescence.

### 1.2 The Views and Voices of Young People: Mental Health Constructs and Measures

Deciding upon the inclusion of mental health constructs, predictive factors and measures is a cornerstone of the research process [8]. There are a variety of methods by which this can be done, including the involvement of ‘experts’. Reflecting increased recognition of the value of including the voices of those affected by research in the research design process, there has been a shift towards prioritising insights made by those with lived experience as a key source of expertise [9].

Given that mental health issues are on a continuum with variation around diagnostic thresholds, the involvement of those with and without experiences of mental ill‐health/use of mental health services is critical [10, 11]. Adolescent involvement has the potential to improve (1) the face validity of outcome measures and (2) the relevance of predictive factors within young people's day‐to‐day and long‐term developmental experiences [12]. This is critical given that one cannot assume a direct alignment between what researchers prioritise and the mental health experiences of young people [13]. Others have pointed to the benefits of co‐development for young people themselves, for example, skill acquisition [14, 15]. Alongside these benefits, a number of authors have emphasised the ethical dimensions of co‐production [16] and the role of democratised decision‐making in promoting equitable relations within research [17, 18]. Finally, the importance of involving those with lived experience has been highlighted by the increased number of publications extolling the virtues of co‐production [19].

Funders also recognise the value of co‐production in mental health research. The UK Research and Innovation's model, as seen in their Mental Health Networks, for example, the Transdisciplinary Research and Innovation for the Improvement of Youth Mental Public Health (TRIUMPH), have emphasised the importance of involving young people as co‐producers from the very beginning of the research cycle [20]. This has also been emphasised by major mental health research funders such as the Wellcome Trust [21] and the National Institute for Health and Care Research [22].

Co‐production is most effective when adolescents are involved in research from the outset. This could include, but is not limited to, (1) developing research questions, (2) deciding upon variable inclusion, for example, outcome, predictors, controls and (3) the tailoring of methodological approaches [15, 23, 24]. An example is the NeurOX YPAG, created to support the development of studies at the intersection of ethics, mental health and technology use [15]. Pavarini et al. found that involving young people led to researchers shifting towards themes/topics deemed more relevant to young people's lives. In addition, the DigiCAT project illustrates the diversity of projects that can adopt a co‐production approach [25]. DigiCAT developed a statistical analysis tool to promote the use of counterfactual analysis in relation to mental health data. Although young people were not the tool's end user, the involvement of young people to support the prioritisation of what they termed ‘active ingredients’, for example, influencing factors, allowed researchers to focus on using the tool to address research questions most relevant to young people.

Mental health research has seen a shift towards developing a common set of measures for research with young people. The International Alliance of Mental Health Research Funders (IAMHRF) has launched a collaborative effort known as the Common Measures in Mental Health Science (CMMHS) initiative, which advocates for the development of a set of measures to promote harmonisation and reproducibility [26, 27]. It is argued that the use of a common set of measures permits (1) the combination of data from across a variety of studies/contexts and (2) the subsequent comparison of outcomes across studies/contexts. The consistent use of a core set can also allow for the examination of sub‐populations which, due to statistical power concerns, are typically left out of studies or are underpowered [27]. Yet it is important to note that a number of criticisms have been made regarding the transferability of measures across cultures [28]. Given this shift towards measurement harmonisation, it is critical that the voices of young people guide the choice of these measures.

An example of this is Power et al. [27] who, through a Delphi methodology, developed a list of mental health measures that can be used for research into neurodivergent young people who have care experience. While there were similarities between the insights of those with lived experience and academics, there were clear disagreements. For instance, the use of the Strength and Difficulties Questionnaire (SDQ) [29] for this population was seen as unproblematic by academics; however, those with lived experience detailed clear flaws related to language use. This is critical given that a recent systematic review [12] posits that mental health outcomes fail to capture relevant concerns, leading to validity issues. Leveraging the insights of those with lived experience, according to the authors, can help researchers make decisions about research mental health outcomes, predictive factors and measures.

Given the above, this paper will examine the insights and recommendations made by the Mental Health in the Moment Young Person Advisory Group (MHIM‐YPAG) members in relation to the inclusion/exclusion of mental health constructs, predictive factors and measures. The goal is for these insights to provide guidance to those engaging in adolescent mental health research with the aim of embedding adolescents' perspectives within subsequent research. We believe that this could be helpful given that not all researchers have the means to fund what is a resource‐intensive process.

### 1.3 Mental Health in the Moment Young Person Advisory Group

The MHIM‐YPAG was established to shape the MHIM project. MHIM is a longitudinal measurement burst study of adolescents in Scotland, examining the interplay between day‐to‐day experiences and adolescents' mental health development (see [30, 31]). This 5‐year project will collect data 6‐monthly, using surveys and ecological momentary assessments (EMA) with 6 prompts each day over 14 days. In addition, biosamples (hair or nail) will be taken to measure stress biomarkers (cortisol). A sub‐sample of participants will have their sleep measured over the study duration through radar‐based technology. Parents/caregivers will also be asked to complete an annual survey to support triangulation. This paper was developed before the first burst of data collection to make sure that members' views were reflected in the choice of constructs, predictive factors and measures.

As outlined within the MHIM‐YPAG protocol [31], engagement was split into two phases: ‘formative’ and ‘data collection’. The first phase, to which the present article relates, focused on defining study parameters. It consisted of four core sessions: (1) mental health constructs/predictive factors, (2) mental health measures, (3) sleep and stress measurement and (4) overall design. By involving young people at this stage, the goal was to embed their voices early on to avoid concerns highlighted above, for example, poor validity and appropriateness. Prior to these sessions, an introduction session was held that focused on ascertaining topic awareness. During that session, we also provided a detailed overview of research, mental health and mental health research.

It is important to stress that this paper reflects insights provided by members and is thus not a traditional research study. Members are an integral part to the decision‐making process and are not ‘participants’. As is reflected in the MHIM‐YPAG protocol, we promote equitable decision‐making whereby members' votes hold greater weight. However, in order to systematically and rigorously gather members' insights, the methods used are associated with traditional research studies.

## 2 Methodology

### 2.1 Members

Age (11–19) and UK residency were the only inclusion criteria with 18 panel places being available. This was to allow a broad range of adolescents to participate. An average of 12 members engaged on a regular basis—both in online sessions and follow‐up surveys. Of these, 8 self‐identify as female, 3 as male and 1 as non‐binary. At the time of recruitment, the age range of members was 11–17 years old. Members identified as Scottish (3), White/White British (3), African (2), Chinese (1), British Indian (1) and European/Caucasian (1)—1 member did not provide this information. In response to members' emphasis on the need for flexible engagement, it was not mandatory for members to attend sessions or submit votes—thus, numbers in the below tables may be inconsistent.

### 2.2 Methods

Researchers undertook an initial review of the literature pertaining to adolescents' mental health, specifically focusing on mental health constructs and associated measures. In addition, an overview of pertinent factors was performed, guided by previous work with young people [24, 26, 32, 33]. This allowed us to develop a ‘long‐list’ of constructs, predictive factors and measures which formed the basis of these sessions. As indicated above, the overall aim of this paper is to provide guidance to prospective researchers and to highlight the benefits of including young people as co‐developers.

### 2.3 Questions Included

- According to young people, what mental health constructs should be prioritised in adolescent mental health research?
- According to young people, what mental health predictive factors should be prioritised in adolescent mental health research?
- According to young people, what mental health measures should be prioritised in adolescent mental health research?

Prior to the mental health constructs and predictive factors session, members were asked to review materials to consider (1) the relevance of the listed mental health constructs/predictive factors for young people and (2) whether there were any mental health constructs/predictive factors missing that should be included. The materials consisted of two tables, one with mental health constructs and one with mental health predictive factors. The former included anxiety, depression, well‐being, quality of life, suicidality/suicide‐ideation, externalising behaviours, eating disorder pathology (EDP) and body dysmorphic disorder (BDD). The latter included academic stress, sleep, physical activity, relationships and social media use. Each table included a description, an example and a pictorial representation. Within this online (Teams) session, members were put into age‐bracketed breakout rooms (11–13; 14–17) to discuss the topics/questions. Following the session, members were asked to complete a survey, which, if they had not done so in the session, asked them to rank the constructs and predictive factors. Sessions were a ‘focus group style’ as this (1) allows for the leveraging of group dynamics to promote nuanced discussion and (2) can act as a means of promoting equitable engagement. Within these sessions, researchers acted as facilitators and, where appropriate, directed conversations to unexplored areas.

A similar approach was taken for the mental health measures session. Members received an information pack which included an overview of two measures for each of the five mental health constructs (see Table 1). All measures had to be free to access to promote reproducibility. Tables within the materials included construct descriptions, a pictorial representation and links to the measures (for viewing purposes). Members were asked, prior to the session, to consider these questions: (1) What are the strengths and weaknesses of each measure? (2) For each mental health construct, which measure do you prefer, if any? And why? (3) Which measures would you include in the study, if any, and which would you exclude? As before, during the session, members were split up into age‐bracketed breakout rooms. Once the session was complete, members were sent a follow‐up survey that asked them to rank each pair of measures.

**Table 1:** Mental health constructs and measures.

Mental health construct	Measure
Anxiety	Revised Children's Anxiety and Depression Scale‐25 (RCADS‐25) [34] Screen for Child Anxiety Related Disorders (SCARED) [35]
Depression	Revised Children's Anxiety and Depression Scale‐25 (RCADS‐25) [34] Short Mood and Feelings Questionnaire (SMFQ) [36]
Quality of life	KIDSCREEN [37] Youth‐Quality of Life‐Short Form Instrument (YQoL‐SF) [38]
Suicidality/suicide‐ideation	Columbia‐Suicide Severity Rating Scale (C‐SSRS) [39] Ask Suicide Screen Questionnaire (ASQ) [40]
Externalising behaviours	Problem Behaviour Frequency Scale (PBFS) [41] Social Behaviour Questionnaire (SBQ) [42]

Once the follow‐up surveys had been completed, a member of the research team (not a MHIM‐YPAG member) reviewed the transcripts and made edits where needed. The same team member then completed a semantic thematic analysis [43, 44], an analysis that was supplemented with notes taken during the session. While these insights may not be typically described as findings, as members are co‐producers and not participants [31], this approach is still useful for distillation. From this analysis, an ‘initial insights’ document that described key emergent themes was developed; this document also included insights from the follow‐up surveys, where members were asked to provide rankings. This document was circulated amongst members and the wider team, who were asked to provide feedback. This verification process was used to assure accuracy. Decisions were made using the jointly established decision‐making protocol. This protocol provides guidelines on how decisions are made within the MHIM‐YPAG, including special circumstances, for example, split decisions, non‐responsiveness and participation below 50%.

While ethical approval was obtained from the University of Edinburgh (reference 253‐2324/11), there is no ethical oversight process available specifically for co‐production. Ethical review for research projects implicitly treats co‐producers as ‘participants’ and can, therefore, undermine efforts to collaborate as equals with young people. Yet, ethical review and oversight is valuable to ensure, for example, informed consent/assent (including parent/caregiver consent), secure information management (e.g., anonymisation of participant contributions and safeguarding of identifiable information) and the appropriateness of compensation (e.g., ensuring that payments are fair but not coercive). In designing our co‐production process, all of these issues were considered. Special consideration was given to the fact that MHIM‐YPAG members are young people and that communication needs to be age‐appropriate to assure understanding. We reiterated their right to discontinue their involvement at any point. In addition, given the sensitive nature of the topics discussed, we also examined the risk of distress from engagement and developed a protocol accordingly. Finally, as members are from a diverse range of communities—including ethnic and sexual minorities—we made sure to engage sensitively and asked members to do the same. To facilitate this, we co‐developed a code of conduct at the start of the process. While helpful, there remains a need for an ethical process dedicated to the particularities of co‐production.

## 3 YPAG Insights

### 3.1 Overview

Overarching insights included the need for both (1) specific (anxiety, depression) and holistic (well‐being, quality‐of‐life) mental health constructs and (2) positive and negative mental health constructs.

In relation to the first point, members appreciated the value of both holistic and specific mental health constructs suggesting that a balance between them allows for greater applicability across the population. For example, while adolescents may not have a specific mental health diagnosis, they are likely to have had periodic experiences of, for example, low mood. Members also emphasised the need for constructs that take into account positive and negative mental health experiences. As articulated by members, mental health is more than a diagnosis or an experience of ill‐health; it is a collection of experiences and feelings that can be both negative and positive. When discussing the quality‐of‐life measures, one member stated:

I like them because it wasn't […] a specific feeling. It was sort of, more of an overall term rather than if you were, like, feeling depressed or feeling anxious. [It's] more just about your well‐being in general and that could be, like, a positive or a negative thing.

When asked to further expand upon the latter point, they stated:

You experience both, like even if somebody had depression there would still be points in their life where they […] would be enjoying themselves or having fun. So, it's important to acknowledge that even if somebody's struggling with mental [ill]‐health, they're not struggling with it constantly. There are times people find enjoyment and have fun.

The above indicates that there are clear benefits associated with using more general/holistic mental health measures, specifically their capacity to capture emotional experiences that lay outside diagnostic parameters. In addition, the ability of measures to assess positive experiences is critical given that, in periods of mental ill‐health, there are moments of reprieve, and it is important to elucidate these ‘protective factors’ [45].

### 3.2 Mental Health Constructs

Table 2 outlines members' ranking of mental health constructs and their use within adolescent mental health research.

**Table 2:** Ranking of mental health constructs.

Construct	Ranking
Anxiety	1st
Depression	2nd
Well‐being	3rd
Quality of life	4th
Behavioural problems	5th
Suicidality/suicide‐ideation	6th
Eating disorder pathology	7th
Body dysmorphic disorder	8th

Members favoured the inclusion of social–emotional disorder constructs, such as anxiety and depression, alongside more holistic constructs, for example, well‐being and quality of life, reflecting insights made above.

While suicidality/suicide‐ideation was ranked low in the follow‐up survey, it drew significant reflection within both sessions. Members emphasised that while suicidality/suicide‐ideation is a sensitive topic, it is not uncommon. Given this, not including it could negatively impact intervention development. While discussing the inclusion of suicidality/suicide‐ideation, one member stated:

I agree that the wording should be quite careful, but I do think that it should be included. Because even though it is quite, like, a dark thing or something that people don't really speak about, it is something that young people experience and it's very serious. So, I think it should be included.

Expanding upon this, another member stated:

I think the first thing is that […] excluding suicide from someone's, like, experience, like not having it be present at all, […] it doesn't give you a whole picture. Specifically, because suicide is often very impactful because it's such an extreme measure, it can be very, I guess, overbearing. And, like, because of that I think the amount you lose, by excluding it, is quite significant. […] if you include it, you have to be not only very careful about how you talk about it, because it can be potentially triggering, but also because […] If there is […] any question that someone will answer dishonestly, it will be a question about whether or not they have experience with suicidal ideation.

The above clearly emphasises the importance of including suicidality/suicide‐ideation within adolescent mental health research and the potential risks of not. This was further emphasised by other members who, reflecting on their personal experience, suggested that this was something that adolescents as young as 11 can experience. However, all agreed that there would need to be careful consideration given to the wording of questions and the length of questionnaires—emphasising age‐appropriateness. This is due to the perceived risk of (1) triggering young people and (2) young people exploring suicidality (online or otherwise) after viewing questions pertaining to it. Finally, members stated that support information should be made available to young people answering these questions. Importantly, within the follow‐up survey, members stated that questions relating to suicidality/suicide‐ideation should be asked to those 11+, but only if this is communicated to parents/caregivers in the information sheets and participants are able to skip these questions.

In terms of additional constructs, members suggested two possibilities: (1) **neurodiversity** and (2) **trauma‐related disorders**.

### 3.3 Mental Health Predictive Factors

Members were also asked to rank factors that are key to adolescent mental health. The factors included within Table 3 differ from those that were originally discussed in the session: an initial list of factors, derived from previous work with young people, was developed. However, during the session, members were asked to indicate factors that they considered missing. These included home life, food/diet, loneliness, identity and sexuality. Thus, Table 3 is a composition of the original list with the additions made by members.

**Table 3:** MHIM‐YPAG ranking of mental health factors.

Factor	Rank
Social media use	1st
Academic stress	2nd
Relationships	3rd
Sleep	4th
Physical activity	5th
Home life	6th
Food/diet	7th
Loneliness	8th
Identity	9th
Sexuality	10th

As shown in Table 3, social media use, academic stress and relationships ranked highest amongst members. In addition, we proceeded to unpack the importance of different types of relationships with members. Table 4 outlines the ranking of these relationship types. As shown, parent/caregiver, friends and romantic partners/other family members ranked the highest.

**Table 4:** Ranking by MHIM‐YPAG members on the impact of different relationships on young people's mental health.

Relationship	Rank
Parent/caregiver	1st
Friends	2nd
Romantic partner/s	Joint 3rd
Other family members	Joint 3rd
Mentors	4th
Teachers/tutors	5th

### 3.4 Mental Health Measures

Key points that emerged out of the session on measures included (1) the length of the measure, (2) the response scale and (3) the choice of language. The younger group (11–13) favoured shorter measures, as it limits fatigue, while the older group (14+) preferred longer measures, as it allowed for a greater depth of understanding. However, in the follow‐up survey, members were asked, in the context of a large, multi‐construct survey, whether a longer measure (SCARED) or a more concise measure (RCADS‐25) should be used to measure anxiety; the majority (88%) of members voted for the latter. Thus, there seemed to be an understanding that, when this context is considered, concerns pertaining to fatigue and overburdening were prioritised.

In addition, in relation to response scales, while there was a difference in opinion, the majority of members, across age groups, advocated for scales with a greater number of options. The reason for this is that it allows individuals to reflect upon their experiences in greater detail. Limiting the options available could lead to answers which are less accurate, questioning the validity of findings. Commenting upon the response scales of measures, members stated:

[…] [it] had, like, more options for replying to it. More options that are just like ‘not true’, ‘sometimes true’, which I think is more, I think [it] is important because then you have more choice.

I think the questions being overly blunt with, like, a ‘yes’ or ‘no’ response, like, that is not only unhelpful, it doesn't give you actual data, but potentially upsetting and dangerous.

Members outlined the risks of response scales deemed too short/choice limiting. The risks are that researchers could be obtaining inaccurate data while simultaneously upsetting participants.

Finally, repeating comments made regarding mental health constructs, members emphasised that there needed to be a greater use of positive language. Commenting upon the potential risks of this, a member stated:

I much preferred, […] just, like, the kind of wording of the second one [measure], I felt it was a lot more, like accessible, I guess, like, more, it seemed more friendly. Whereas this is really quite negative. […] If I was to fill that out, I'd feel quite down at the end of it, whereas the other one, it wasn't sort of like that. It was more, it was more open and less sort of like [negative]. […]. For, like, the study, […] you know what you're getting into when you're setting up for the study, but constantly having to review these types of feelings could […] have a negative impact on somebody's mental health. So, I just, I preferred the wording of the other question because it's not as negative. Like you'd still get the results that you'd want about whether somebody was feeling anxious or depressed, but without, like the young person sort of feeling like, ‘oh, there must be something really wrong with me’.

The above points to the potential risks to participants if negatively orientated measures are used. In addition, reflecting upon both measures, members stated that utilising a measure which includes a focus on positive mental health experiences does not limit its ability to capture anxious/depressive symptoms.

Moreover, some of the members, specifically those from the older group, commented upon how the language used was at times condescending, infantilising and insensitive. For instance, one member stated:

I find the language […] a little bit patronising and condescending. Like it's… I agree it has ‘bad vibe’. I guess [that's] where I'm coming from. […] I do feel this one comes across as a little condescending, as if it's talking down to me.

This member further expanded upon their statement by suggesting that the condescending nature of the language used could lead to them not answering truthfully. Given this, researchers need to closely consider the language used in measures, its applicability across the adolescent age group and the value of alternative measures.

Table 5 details the outcome from the measure ranking. The ‘number of votes’ is an aggregate of both age groups. Differences in the number of votes across measures reflect the fact that some of this voting took place in the session, while in other cases it occurred within the follow‐up survey—it was not mandatory for members to cast their vote in either the session or the survey.

**Table 5:** MHIM‐YPAG voting on the mental health measures.

Construct‐ measure	Number of votes
Anxiety‐ RCADS‐25	8
Anxiety‐ SCARED	1
Depression‐ RCADS‐25	5
Depression‐ SMFQ	3
Quality of life‐ KIDSCREEEN	2
Quality of life‐ YQoL‐SF	8
Externalising behaviours‐ PBFS	5
Externalising behaviours‐ SBQ	3
Suicidality‐C‐ SSRS	6
Suicidality‐ ASQ	2

In relation to the use of RCADS‐25 for anxiety, members initially preferred SCARED citing concerns that RCADS‐25 used overly negative language while SCARED was seen as being more accessible, age‐appropriate and less infantilising. In addition, due to its length, members suggested that this allowed an appreciation of nuance and would reduce the chance of misdiagnosis. However, when reminded that this would be a multi‐measure survey, members chose RCADS‐25 due to the risk of fatigue. For depression, RCADS‐25 was favoured due to its length, variety of questions and scaling. While the SMFQ was seen as oversimplifying depression and failing to capture its complexity.

In relation to the quality‐of‐life measures, the majority preferred the YQoL‐SF due to its larger response scale, permitting a greater depth in understanding, and its relatability, as it focused on personal experiences. While KIDSCREEN was seen as accessible and appropriate for all ages, a member suggested it lacked overall specificity. In addition, some members felt the wording to be inappropriate and not reflecting the diversity in familial arrangements, for example, parent compared to foster parent.

For externalising behaviours, members preferred the PBFS due to its use of appropriate, simple and relevant language. However, it would need cultural adaptation, for example, ‘spirits’ to replace ‘liquor’ in a British context. In addition, members suggested that ‘vapes’ should be included within this measure. They stressed the importance of a measure that incorporated both perpetrator and victim questions.

Lastly, pertaining to suicidality, while the length of the ASQ was liked, specifically amongst the younger group, the overall opinion was that there were clear issues with language. For instance, the use of a binary answering scale within the ASQ was seen as limiting and thus failing to account for nuance. Members also suggested that a number of questions were insensitive and could cause distress if not handled professionally. Moreover, while they preferred the C‐SSRS, there were still problems. Members stated that both measures could pose a risk to participants, specifically in studies that use remote data collection methods (such as smartphone‐based surveys/EMA, etc.) where participants are answering questions independently. Thus, while there was a preference, members did not recommend either measure.

## 4 Discussion

The involvement of young people within research has become a priority for the field, leading to the proliferation of participative methods [27, 46, 47, 48]. This paper focused on documenting the insights offered by MHIM‐YPAG members regarding mental health constructs, predictive factors and measures. We believe this will support researchers within the field to make project parameter decisions. In addition, by detailing member priorities and rationales, we also aim to highlight the benefits of co‐production.

In the case of the MHIM study, this led to the inclusion of specific constructs, predictive factors and the selection of measures. While some of these were already short‐listed for inclusion, other measures were added as a result of member insights. The process helped make the study more applicable to young people's experiences. As noted above, while these insights are not meant to be generalisable across time and space, we believe that researchers could draw from these insights when co‐developing their own projects.

In terms of mental health constructs, members ranked anxiety and depression highest in relevance, consistent with prevalence rates during adolescence (4.1%–5.3% for anxiety and 1.3%–3.4% for depression), when compared with EDP and BDD (0.1%–0.4%) [49, 50]. Members also emphasised the necessity of balancing specificity and generality, that is, specific diagnoses (anxiety/depression) and holistic constructs (quality‐of‐life/well‐being). This was also consistent with a previous Delphi study [27]. Within their study, young people emphasised that diagnostic constructs, while useful, could be limiting due to their failure to account for general emotional experiences.

Finally, members highlighted that consideration must be given to sensitive constructs, for example, suicidality. While there may be perceived ‘risks’ associated with investigating such topics, a failure to include them is a failure to effectively understand adolescents' mental health. For instance, as detailed by Mars et al. [51], suicide‐ideation in adolescence is common and highly correlated with suicide behaviours [52, 53]. Mars et al. further argue that due to this relationship, understanding the link between ideation and behaviours is critical to developing interventions. However, as outlined by members, the way in which this is done is of critical importance [54, 55]. Lastly, while the language used is important, research tends to show that asking about suicidality does not increase the risk of young people engaging in those behaviours; in fact, it can lead to the opposite [56].

Pertaining to predictive factors, the ranking of social media as first was, again, consistent given its dominance within public discourse. For instance, the Scottish Government, alongside other countries such as Australia, the Netherlands and South Korea, has recommended the limiting of mobile devices in schools [57]. While there is evidence of the negative relationship between social media use and adolescents' mental health [58, 59], the pattern of this relationship is nuanced [60]. Specifically, the link between social media use as measured by ‘screen time’ is weak to null when adjusting for confounders [61]; see also [62, 63]. Yet evidence suggests that specific online activities such as engaging in social comparison may be damaging for mental health [64].

Moreover, members commented upon the value of including academic stress as a predictive factor. This is unsurprising considering that (1) adolescents typically spend the majority of this period at school and (2) pivotal academic milestones, for example, exams, become more frequent [65]. Guo et al. [66], for instance, found that increases in academic stress were associated with increased depressive symptoms at age 15 (see also [67]) and related to an 8% higher odds of self‐harm.

Members also highlighted the importance of relationships within young person's lives—an association sustained within the literature [68]. Studies have looked at the different types of relationships and their impact on mental health trajectories [69, 70, 71, 72, 73, 74]. Importantly, Table 4 can act as a guide for researchers interested in relational impacts. Given the importance in understanding how relational dynamics impact a young person's mental health, the MHIM study will examine daily relational experiences and their influence on mental health development, including all of the relationships in Table 4 in the survey measures and the top‐ranked ones in the EMA component.

In terms of measures, members pointed to a preference for measures that use (1) appropriate language (non‐triggering and non‐infantilising) [75], (2) response scales with more options [76, 77, 78], (3) positive and negative construct language, for example, a strengths‐based approach [79] and (4) measures that are specific and holistic [27]. Given this, it is critical that adolescents, across the age range of a study, are involved in (1) the selection, or where necessary, development and validation of these measures and (2) providing an assessment of language appropriateness and measurement face validity. Critically, the measures examined in the current co‐production exercise can be used across a wide age range, for example, 11–18, thus a close consideration of how the language is interpreted across this range is important.

## 5 Challenges

This paper points to the value in using participatory methods in adolescents' mental health research. However, there are clear challenges, for example, diminishing engagement. In relation to limiting this issue, we recommend that researchers develop (1) flexible engagement methods, (2) regular contact periods, (3) timescales for group decisions, outlined in a ‘decision protocol’ and (4) an incentive structure that is not limited to monetary reimbursement. Further research should examine methods of maintaining engagement for adolescent co‐producers.

Other challenges include the adoption of methods that gather robust insights without reproducing the associated researcher/participant divide. Related to this is the need for ethical processes which are tailored to the needs of co‐production and do not treat them as participants but as collaborators. In addition, the process of balancing flexibility with sufficient representation can be difficult. Finally, the need to manage conflicting views/group dynamics and the need to train members so they can meaningfully engage is a challenge.

## 6 Limitations

A key limitation relates to the number of young people involved in the MHIM‐YPAG. Resource constraints limited what could be done to prevent attrition and increase the diversity of voices—critical given that young people cannot be seen as representing an entire population [80]. This also relates to the consistency of engagement. While members were reimbursed for their time, engagement and survey response rate consistency was an issue. Another important limitation is the number of constructs and measures which were examined by members. Due to resource and timeframe constraints, the number which members were able to examine was limited. Given this, we recommend that future studies ensure that sufficient budget is allocated to the co‐production process to allow sufficient breadth and depth of YPAG engagement in construct/measure selection.

## 7 Conclusion

The paper highlights the importance of involving young people as co‐producers when deciding key study parameters. The benefits of doing so include improving the appropriateness of research designs and the overall relevance of the study. While not claiming generalisability, the above insights on which constructs, predictive factors, and measures young people view as most relevant/appropriate may act as guidance for researchers within this area.

## Author Contributions

**Luke Power:** conceptualization, methodology, writing – original draft, writing – review and editing, investigation. **Dejla Hoxha:** writing – review and editing, investigation, conceptualization. **Lorna Caddick:** conceptualization, investigation, writing – review and editing, validation. **Tong Xie:** conceptualization, writing – review and editing. **Hannah Coulstock:** investigation, writing – review and editing. **Alex Crocker:** investigation, writing – review and editing. **Katie Dryburgh:** investigation, writing – review and editing. **Daria Melashenko:** investigation, writing – review and editing. **Dacian Astilean‐Style:** investigation, writing – review and editing, conceptualization, validation. **Heather Bryson:** investigation, writing – review and editing, conceptualization, validation. **Eva Drummond:** conceptualization, writing – review and editing, investigation, validation. **Sharon Ehimen:** writing – review and editing, investigation, conceptualization, validation. **Poppy Fairbairn:** conceptualization, investigation, validation, writing – review and editing. **Rowan Kelly:** investigation, conceptualization, validation. **Paige Sinclair:** conceptualization, investigation, validation, writing – review and editing. **Yu‐Wen Tan:** conceptualization, investigation, writing – review and editing, validation. **Aja Murray:** funding acquisition, writing – review and editing, conceptualization.

## Conflicts of Interest

The authors declare no conflicts of interest.

## Data Availability

The data that support the findings of this study are available from the corresponding author upon reasonable request.
